# *IL1RN* and *PRRX1* as a Prognostic Biomarker Correlated with Immune Infiltrates in Colorectal Cancer: Evidence from Bioinformatic Analysis

**DOI:** 10.1155/2022/2723264

**Published:** 2022-11-29

**Authors:** Qi Wang, Xufeng Huang, Shujing Zhou, Yuntao Ding, Huizhi Wang, Weiye Jiang, Min Xu

**Affiliations:** ^1^Department of Gastroenterology, Affiliated Hospital of Jiangsu University, Jiangsu University, Zhenjiang, China; ^2^Faculty of Dentistry, University of Debrecen, Debrecen, Hungary; ^3^Faculty of Medicine, University of Debrecen, Debrecen, Hungary

## Abstract

The extensive morbidity of colorectal cancer (CRC) and the inferior prognosis of terminal CRC urgently call for reliable prognostic biomarkers. For this, we identified 704 differentially expressed genes (DEGs) by intersecting three datasets, GSE41328, GSE37364, and GSE15960 from Gene Expression Omnibus database, to maximize the accuracy of the results. Preliminary analysis of the DEGs was then performed using online gene analysis datasets, such as DAVID, UCSC Cancer Genome Browser, CBioPortal, STRING, and UCSC Cancer Genome Browser. Cytoscape was utilized to visualize the protein perception interaction network of DEGs, and the bubble map of GO and KEGG enrichment function was demonstrated using the R package. The Molecular Complex Detection (MCODE), Biological Network Gene Oncology (BiNGO) plug-in in Cytoscape, was applied to further screen the DEGs to obtain 15 seed genes, which were *IL1RN*, *GALNT12*, *ADH6*, *SCN7A*, *CXCL1*, *FGF18*, *SOX9*, *ACACB*, *PRRX1*, *MZB1*, *SLC22A3*, *CNNM4*, *LY6E*, *IFITM2*, and *GDPD3*. Among them, *IL1RN*, *ADH6*, *SCN7A*, *ACACB*, *MZB1*, and *GDPD3* exhibited statistically significant survival differences, whereas limited studies were conducted in CRC. Based on the enrichment results of the “Gene Ontology“(GO) and “Kyoto Encyclopedia of Genes and genomes “(KEGG) as well as documented findings of key genes, we further emphasized the potential of *IL1RN* and *PRRX1* as markers of immune infiltrates in CRC and confirmed our hypothesis by compiling data from the UALCAN, Tumor Immune Estimation Resource, and TISIDB databases for these two genes. The above-mentioned genes might offer a valuable insight into the diagnosis, immunotherapeutic targets, and prognosis of CRC.

## 1. Introduction

Colorectal cancer (CRC) remains the third most prevalent cancer globally, with the most recent data estimating its incidence to be the second highest mortality rate [1]. Smoking, processed meat, alcohol intake, red meat, low intake of vegetables and fruits, body fat, and obesity were all identified as risk factors for the pathogenesis of CRC [2, 3]. Despite advances in combination treatment regimens and individualized therapeutic planning over the past decade, the average survival time for advanced CRC has improved significantly [4]. However, compared to a 5-year survival rate of approximately 90% for patients with early-stage CRC, the 5-year survival rate of those with advanced distant metastases has fallen to less than 10%, suggesting that earlier diagnosis and treatment are key to effectively optimizing the prognosis of patients with CRC as well as reducing the burden of disease in the population [5]. Invasive and semi-invasive screening modalities are effective in detecting early CRC, yet studies point out that the overall screening rate for CRC does not reach the expected results, and few interventions are proven to increase the acceptance of such screening [6]. Both precise treatment and early screening for CRC call for more non-invasive early screening biomarkers as well as staging prognostic markers based on a deeper understanding of the pathogenesis of CRC [7]. Therefore, great interest still exists to further innovate the methodology in early diagnosis of CRC.

Biomarkers are signposts for early cancer detection and individualized CRC treatment [8]. Most notably, not only do KRAS mutations accompanied with high risk of recurrence and metastasis after radical resection of CRC, but also suggest a poorer overall prognosis after resection of liver metastases from metastatic CRC [9, 10]. Carcinoembryonic antigen (CEA) may be the most widely used clinical biomarker to predict early recurrence in postoperative patients [11]. Nevertheless, for CRC, its sensitivity and specificity of the test are low. Exploration of biomarkers that enable reliable estimation of CRC prognosis might provide far-reaching implications in supporting therapy of CRC [12]. Current bioinformatic techniques to identify molecular targets that serve as biomarkers for CRC constitute the mainstream research approach [13]. Owing to genomic profiling methods coupled with updating bioinformatics algorithms, comprehensive data association in combination with bioinformatics analysis has enabled the identification of plenty of clinical biomarkers available for non-invasive cancer screening and prognostic assessment of oncology patients [14]. Meanwhile, sophisticated mechanisms within the tumor microenvironment (TME) appear to be an emerging factor influencing the prognosis of patients with malignancies, with the consequent recognition that tumor-infiltrating immune cells and tumor-associated stromal cells can greatly impact on tumor progression and clinical outcome [15]. Identifying markers indicating the intricacies of the CRC TME has been a hot trend in bioinformatics, with immune-related prognostic genes being of considerable significance [16].

Hence, here, we obtained the differentially expressed genes (DEGs) of CRC by interacting multiple Gene Expression Omnibus (GEO) gene microarray datasets, furthermore, using diverse bioinformatics tools (DAVID and Tumor Immune Estimation Resource [TIMER]) to explore the possible mechanisms linking DEGs to CRC. These include GO and KEGG pathway enrichment analyses, and protein perception interactions (PPI) network as well as immune infiltration were also included. It also reveals the potential critical role of *IL1RN* and *PRRX1* in CRC. Furthermore, mapping of DEGS function enabled us to identify more precise key genes accompanying with a deeper perception of the role of *IL1RN* and *PRRX1* in CRC.

## 2. Methods

GEO (http://www.ncbi.nlm.nih.gov/geo) database is known as a freely accessible database containing gene expression information, such as gene chips, high-throughput gene expression data, and gene microarrays [17]. GSE41328 [18], GSE37364 [19], and GSE15960 [20] were selected from the GEO database. GSE41328 [18] contains 10 paired CRC and normal tissue samples, whereas GSE15960 [20] contains 6 paired CRC and normal tissue samples. The information regarding the selected CRC tissue samples was not further explained in GSE41328 and GSE15960. 27 CRC tissue samples including 14 cases of colorectal adenocarcinoma at Dukes A/B stage and 13 colorectal adenocarcinoma at Dukes C/D stage and 38 normal tissue samples were selected from GSE37364 [19] dataset.

### 2.1. Method 1

Comparison of target datasets filtered from GEO database was performed by GEO2R (https://www.ncbi.nlm.nih.gov/geo/geo2r) [17]. Furthermore, filter terms were set at *P* < 0.05, logFC (fold change) >1 or logFC <−1 for DEG screening [21]. During this process, strict normalization was conducted by using the official tools offered by the GEO database. The intersecting DEGs were obtained by taking three-way intersections of the screened DEGs using the online open access Venn diagram tool (http://bioinformatics.psb.ugent.be/beg/tools/venn-diagrams) [22].

### 2.2. Method 2

GO [23] and KEGG [24, 25] enrichment analyses were constructed by utilizing the DAVID online gene analysis database (DAVID; http://david.ncifcrf.gov) [26], which covers molecular function (MF), cellular composition (CC), and biological processes (BP) [23, 27]. The results of the analysis are visualized in bubble charts by R package (ggplot2), with an adjusted *P* < 0.05 considered statistically significant [8, 28].

### 2.3. Method 3

The DEG interactions were analyzed using STRING (STRING; http://string-db.org), an online gene interaction database, and the PPI interactions network was constructed using a “combined score > 0.4” as a screening condition [29]. The results of PPI interactions were imported into the open access bioinformatics software platform Cytoscape (http://www.cytoscape.org; version 3.6.1) for visualization [30]. The core modules and pivotal prognostic genes among PPI network were screened by MCODE in Cytoscape software [31]. The screening criteria were set with MCODE score >5, degree-cuff = 2, node score = 0.2, max depth = 100, and *k*Score = 2. The major functions of the seed genes were searched via NCBI (https://www.ncbi.nlm.nih.gov/gene) and were presented in a table format.

### 2.4. Method 4

GO enrichment analysis and KEGG pathway analysis of the hub gene were performed through the DAVID Gene Analysis Online database (DAVID; http://david.ncifcrf.gov) [26]. The analysis and visualization of the BP of seed genes were conducted by the Cytoscape Biological Network Gene Oncology plugin (BiNGO) [32]. Construction of hierarchical aggregations of seed genes was undertaken at the UCSC Cancer Genome Browser (http://genome-cancer.ucsc.edu) [33]. Overall survival analysis of hub genes were performed by means of Kaplan-Meier curves in cBioPortal (https://www.cbioportal.org/) [34].

### 2.5. Method 5

UALCAN (http://ualcan.path.uab.edu/), an online data site that integrates RNA-seq and clinical data from 31 malignancies of TCGA (https://portal.gdc.cancer.gov/), was used to perform the analysis of *IL1RN* expression levels with *PRRX1*, including differences in tissue type (healthy/tumor), and GC staging (stages 1, 2, 3, and 4) in COAD as well as READ [35].

### 2.6. Method 6

The TIMER2.0 (https://cistrome.shinyapps.io/timer/) was used to systematically analyze the level of immune infiltration in various malignancies [36]. The relationship between *IL1RN* and *PRRX1* expression and tumor-infiltrating lymphocytes (TILs) expression in COAD and READ was investigated through the TIMER gene module. Furthermore, the interaction between CRC and the immune system, immune cells, was studied through the online platform TISIDB (http://cis.hku.hk/TISIDB/index.php) [37]. This includes the association of *IL1RN* and *PRRX1* with 28 TILs, 45 immunostimulants, and 24 immunosuppressive agents in CRC.

### 2.7. Method 7

The HPA database (https://www.proteinatlas.org/) allows online access to human proteins mapped in cells, tissues, and organs through the integration of a variety of histological techniques (including antibody-based imaging and transcriptomics) [38]. *IL1RN* and *PRRX1* expressions in normal colorectal tissues and CRC tissues were retrieved from the HPA database.

## 3. Results

### 3.1. Result 1

**Figure 1:**
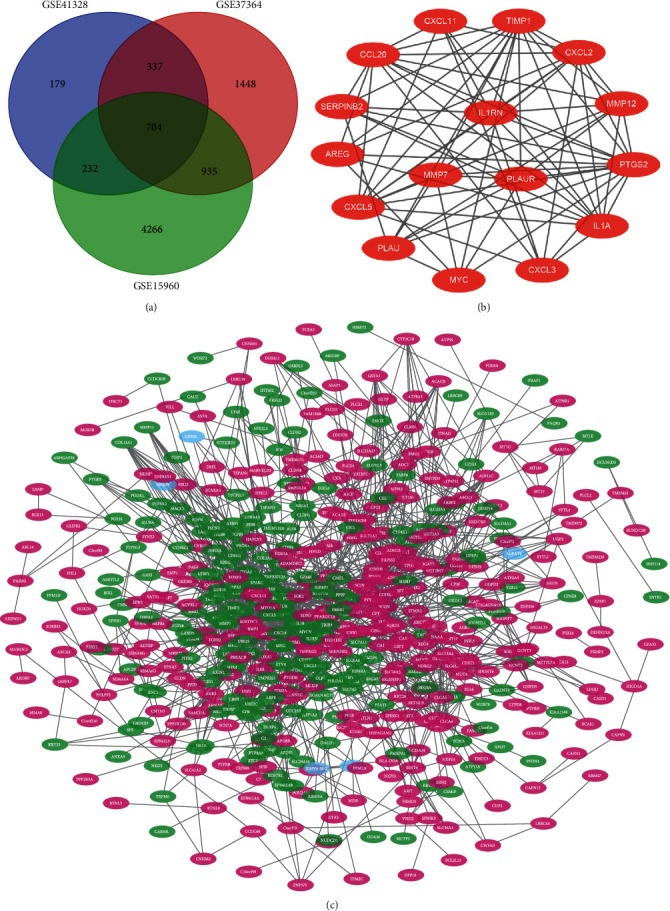
Venn diagram, PPI network, and module of IL1RN. (a) DEGs were selected with a fold change >1 and *P* < 0.01 among the mRNA expression profiling sets GSE41328, GSE37364, and GSE15960. (b) The PPI network of DEGs was constructed using Cytoscape. (c) The most module of IL1RN was obtained from PPI network with 33 edges.

After data validation analysis, 1452 (GSE41328), 3424 (GSE37364), and 6037 (GSE15960) DEGSs were obtained, and a total of 704 DEGs were found to be present in all three datasets using online Venn diagram analysis (Figure 1(a)).

### 3.2. Result 2

STRING and Cytoscape were used to construct a PPI network and a gene perception network of 704 DEGs (Figure 1(b)), both of which clearly showed the presence of dense regions, that is, modules of genes closely related to CRC (key genes). This network consists of 549 nodes and 1740 edges. Applying MCODE to construct the hub genes module, for which the most intensively interacting block seed gene was *IL1RN* (Figure 1(c)) and separating the 15 clusters (edges >5) of seeds genes further yielded 15 key genes *IL1RN*, *GALNT12*, *ADH6*, *SCN7A*, *CXCL1*, *FGF18*, *SOX9*, *ACACB*, *PRRX1*, *MZB1*, *SLC22A3*, *CNNM4*, *LY6E*, *IFITM2*, and *GDPD3*. Detailed information on these seed genes is contained in Table 1.

### 3.3. Result 3

**Figure 2:**
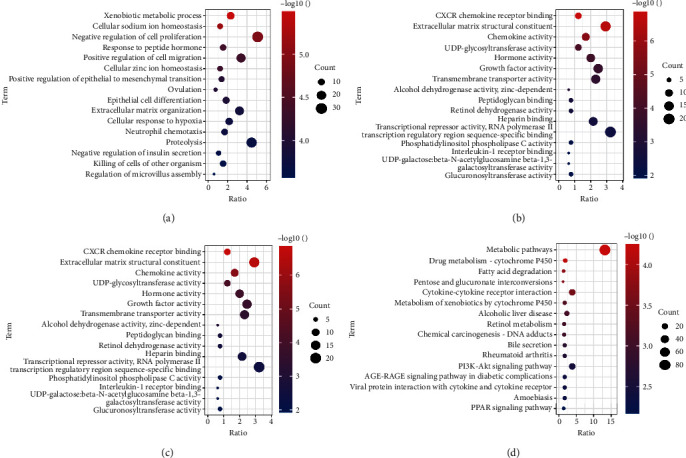
GO enrichment and KEGG pathway functional enrichment analyses of the DEGs. (a) The biological process (BP) of GO classification. (b) The cell component (CC) of GO classification. (c) The molecular function (MF) of GO classification. (d) KEGG pathway functional classification and annotation.

The results of GO enrichment analysis and KEGG pathway analysis of DEGs by the DAVID online tool are presented in bubble charts, sorted by the number of enriched genes, with the top 16 positions. As shown in the figure, the main MFs involved in DEGs are extracellular matrix structural constituent, RNA polymerase II transcription regulatory region sequence-specific binding. DEGs are involved in CC mainly in the plasma membrane, integral component of membrane, negative regulation of cell proliferation, positive regulation of cell migration, and other BP. KEGG results show that DEGs are strongly associated with metabolic pathways and cytokine–cytokine receptor interaction (Figures 2(a), 2(b), 2(c), and 2(d)).

### 3.4. Result 4

**Figure 3:**
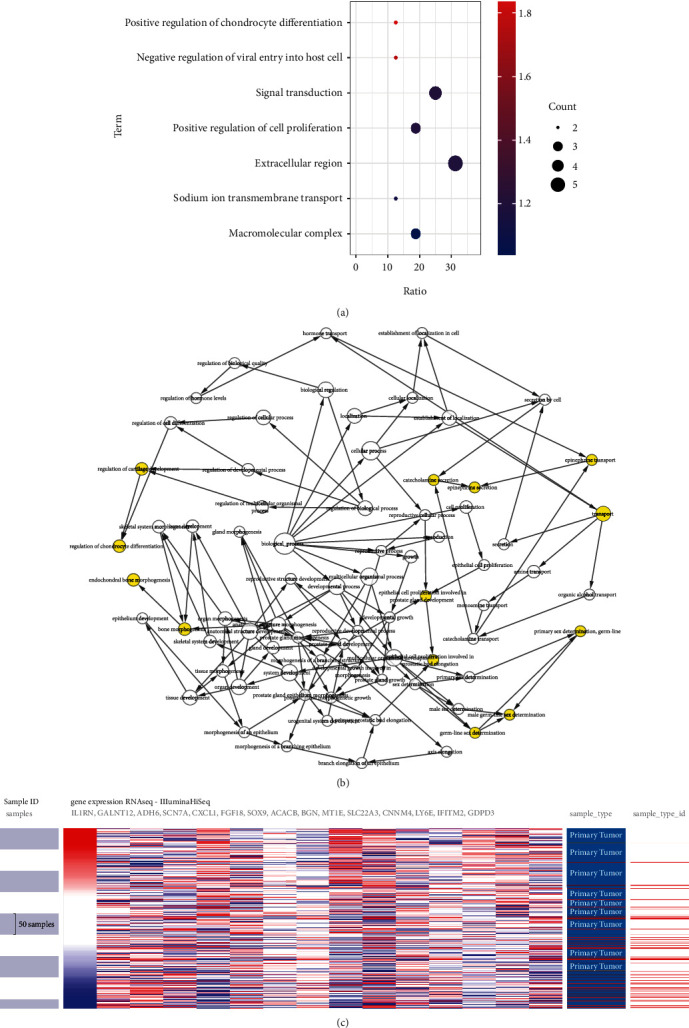
GO enrichment, interaction network, and biological process analyses of the seed genes. (a) BP, MF, and CC of seed genes. (b) The BP analysis of hub genes was constructed using BiNGO; *P* < 0.01 was considered statistically significant. (c) Hierarchical clustering of hub genes was constructed using UCSC. The samples under the pink bar are non-cancerous samples, and the samples under the blue bar are HCC samples. Upregulation of genes is marked in red; downregulation of genes is marked in blue.

**Figure 4:**
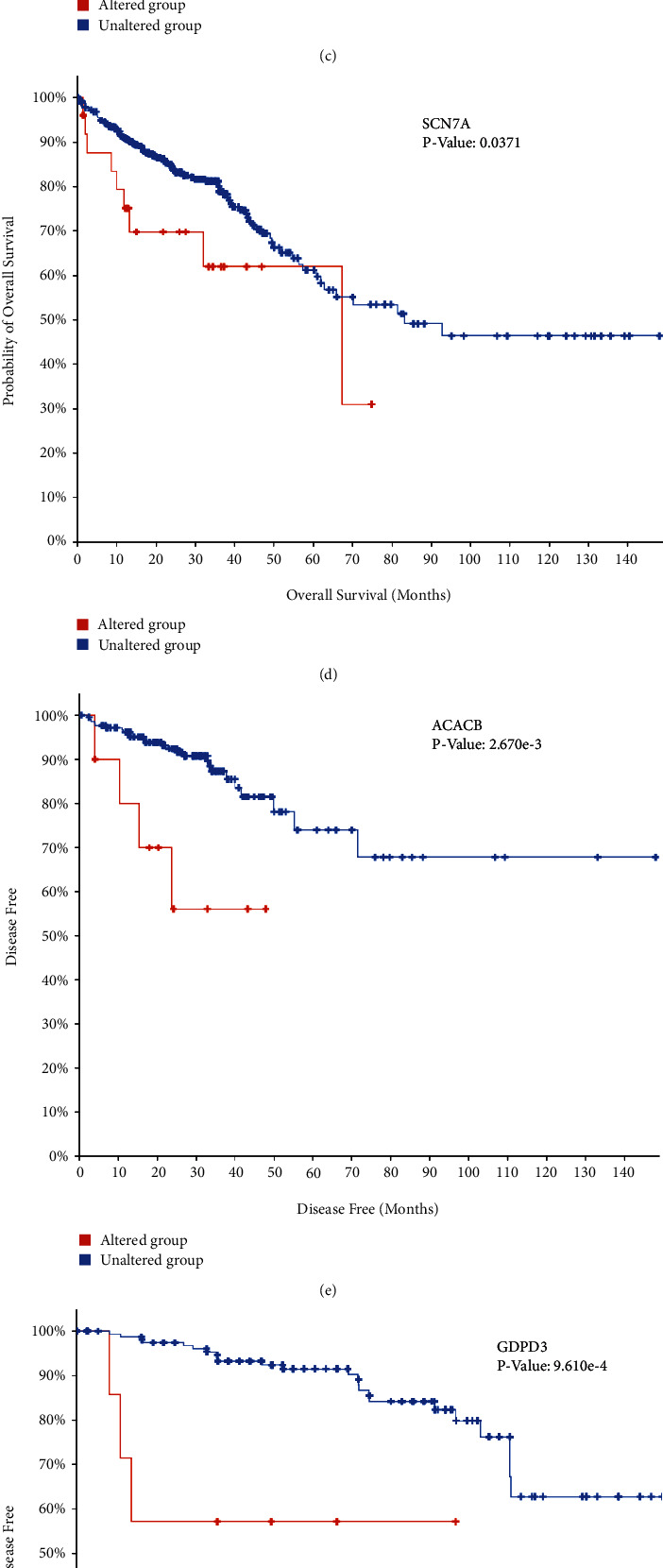
(a)–(d) and (g) Overall survival and (e), (f), and (h) disease-free survival analyses of hub genes were performed using cBioPortal online platform. *P* < 0.05 was considered statistically significant.

The analysis of the biological interacting process of the seed genes is presented in (Figure 3(b)). By applying hierarchical clustering analysis, it allows to judge that the seed gene could clearly distinguish between CRC and normal samples (Figure 3(c)). The function of seed genes was analyzed using DAVID. The results demonstrated that the gene functions in this module were mainly enriched in extracellular region, signal transduction. KEGG results indicated that seed genes are involved in alcoholic liver disease pathological process and pyruvate metabolism (Figure 3(a)). By sorting the results of the CBioPortal database survival data and conducting prognostic survival analysis, it was found that four of the key genes, including *ACACB*, *GDPD3*, *MZB1*, and *SCN7A*, were significantly associated with overall CRC survival (Figures 4(a), 4(b), 4(c), and 4(d)). The correlation between *ACACB*, *GDPD3D*, and CRC disease-free survival is statistically significant (Figures 4(e) and 4(f)). *IL1RN* and *PRRX1* showed higher 33 edges and 20 edges in the PPI network, but disease-free survival of *IL1RN* along with overall survival and disease-free survival of *PRRX1* did not show a statistical difference, although it could reflect an associated correlation with CRC progression (Figures 4(g) and 4(h)).

### 3.5. Result 5

**Figure 5:**
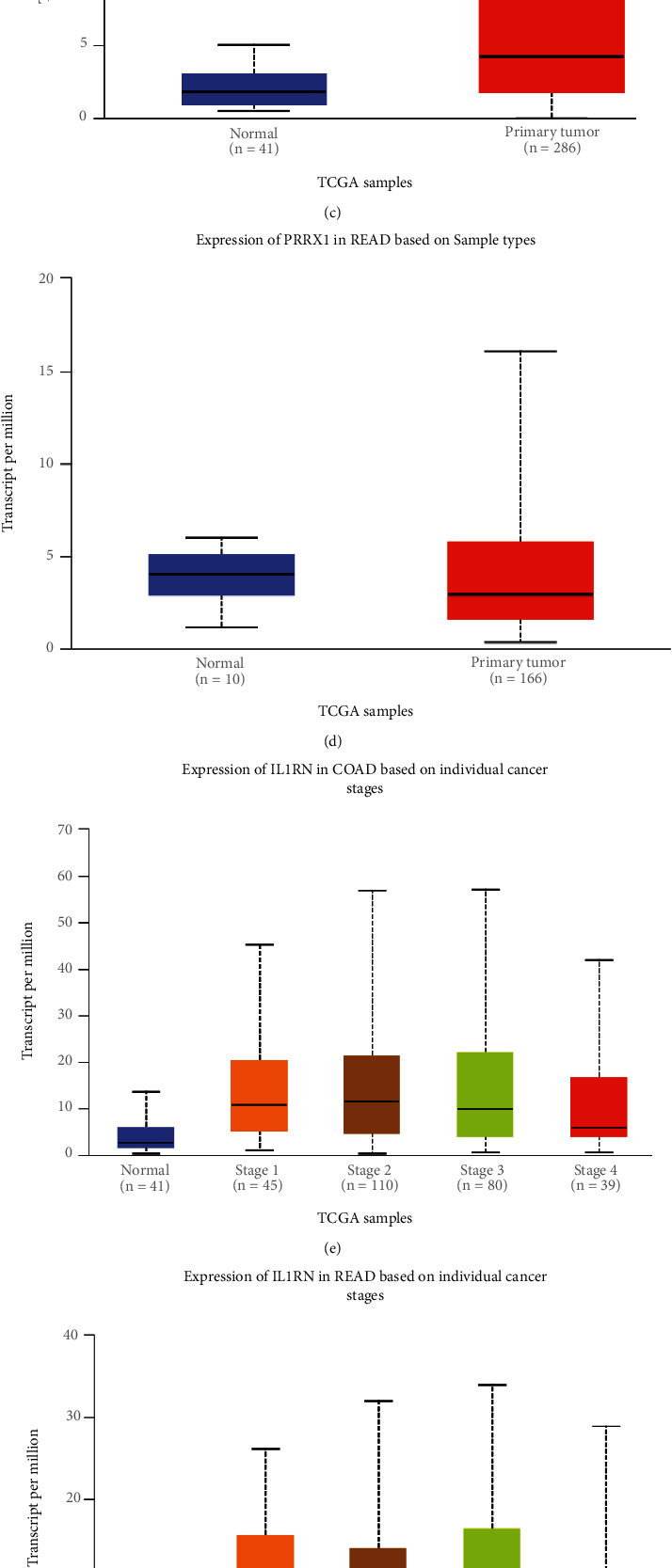
Correlation between *IL1RN* and *PRRX1* mRNA expression level and clinicopathological parameters of CRC through the UALCAN database. (a)–(d) Sample type (normal/primary tumor). (e)–(h) Cancer stage (stages 1, 2, 3, and 4).

The results of UALCAN analysis showed that the expression levels of both *IL1RN* and *PRRX1* were significantly higher in colon and rectal cancer samples than in healthy samples (Figures 5(a), 5(b), 5(c), 5(d), 5(e), 5(f), 5(g), and 5(h)). In addition, this disparity became more obvious with the progression of tumor stage, suggesting a potential function of IL1RN and PRRX1 in tumor development and migration.

### 3.6. Result 6

**Figure 6:**
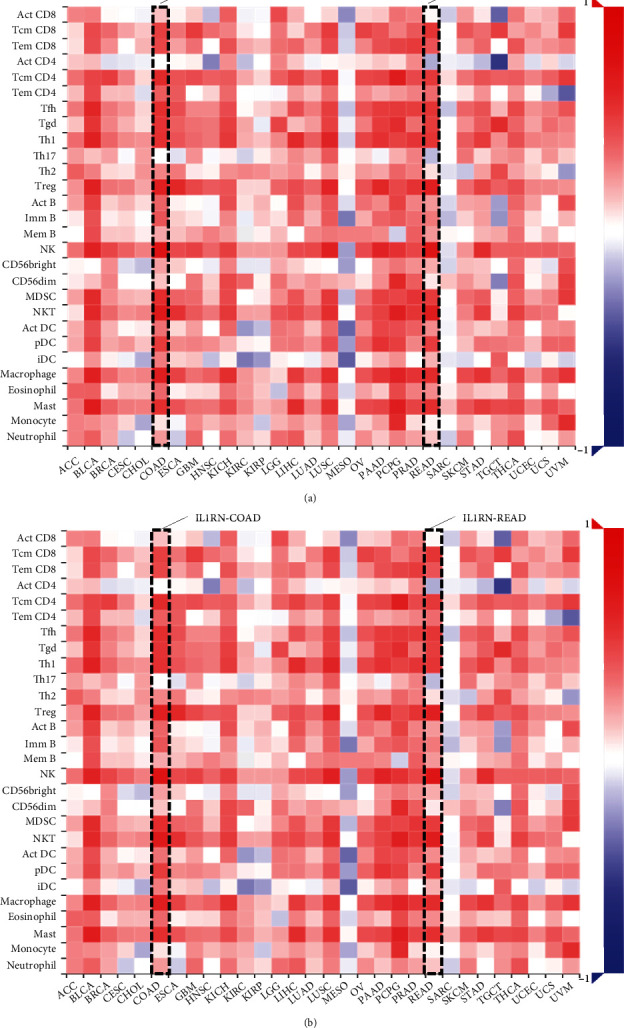
Correlation of *IL1RN* and *PRRX1* expression with immune infiltration in CRC. (a) and (b) Correlation between the expression of *IL1RN* and *PRRX1* and the abundance of TILs in CRC available at TISIDB database.

**Figure 7:**
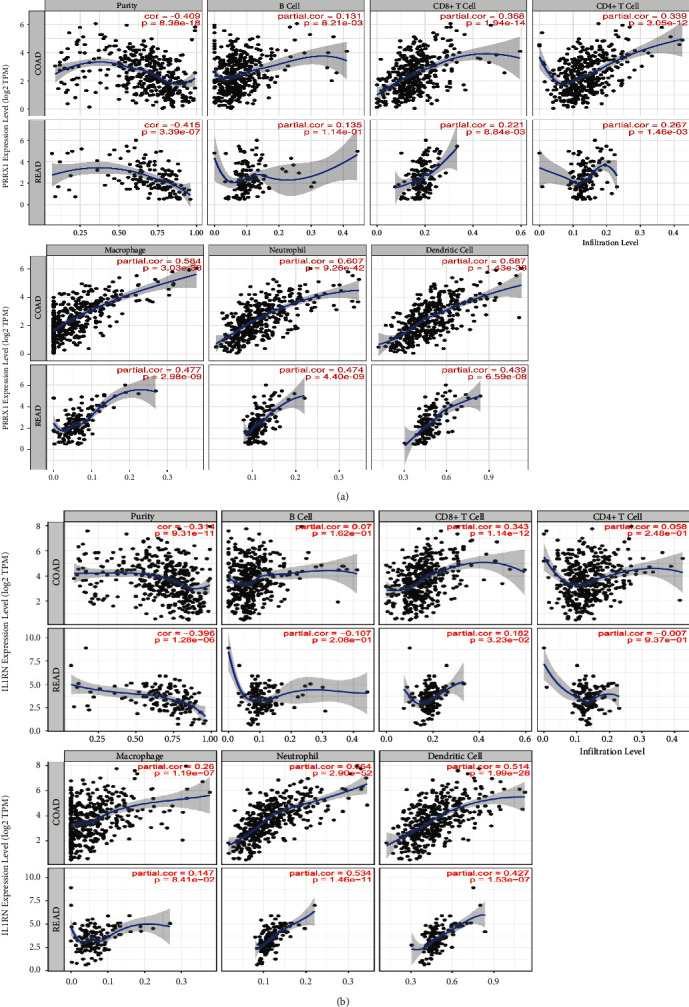
Correlation of *IL1RN* and *PRRX1* expression with immune infiltration in CRC. (a) and (b) Correlation of *IL1RN* and *PRRX1* expression with infiltration levels of TILs CRC available at TIMER2.0 database. TILs, tumor-infiltrating lymphocytes; TIMER2.0, Tumor Immune Estimation Resource.

**Figure 8:**
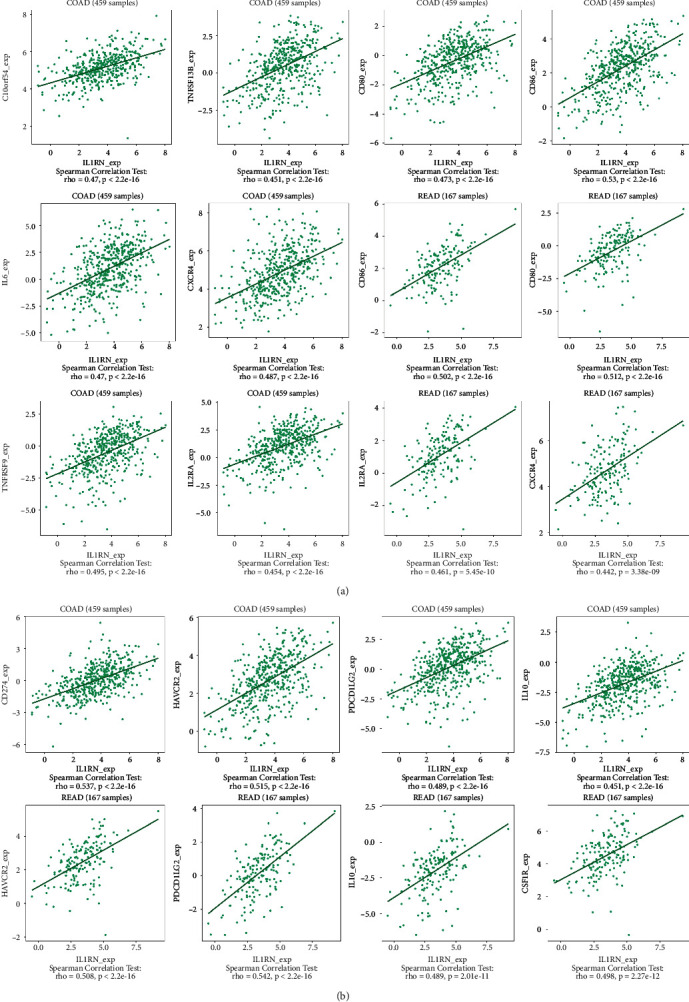
The expression of IL1RN is associated with immunomodulators in CRC. (a) Correlation between *IL1RN* expression and immunoinhibitors in CRC available at TISIDB database. (b) Correlation between *IL1RN* expression and immunostimulators in CRC available at TISIDB database.

**Figure 9:**
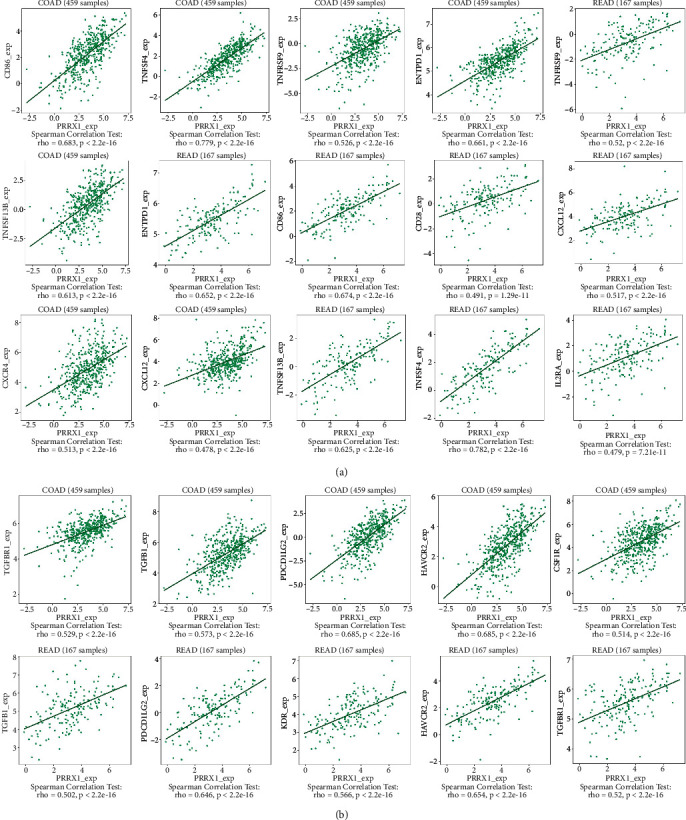
The expression of *PRRX1* is associated with immunomodulators in CRC. (a) Correlation between *PRRX1* expression and immunoinhibitors in CRC available at TISIDB database. (b) Correlation between *PRRX1* expression and immunostimulators in CRC available at TISIDB database.

*IL1RN* belongs to the interleukin 1 cytokine family, with its aberrant expression telling the incidence of carcinogenesis and immunomodulation. *PRRX1* is well established as closely linked with the EMT in malignancies. However, the relationship between *IL1RN* and *PRRX1* in CRC with TILs is unclear. The correlation between the level of immune infiltration of these two in CRC was assessed by the TIMER2.0 database, demonstrating that either *IL1RN* or *PRRX1* correlates markedly with an elevation in TILs (Figure 6). This was reflected by the fact that CD4+ T cells (Rho = 0.339, COAD; Rho = 0.267, READ), neutrophils (Rho = 0.607, COAD; Rho = 0.474, READ), macrophages (Rho = 0.584, COAD; Rho = 0.477, READ), and myeloid dendritic cells (Rho = 0.587, COAD; Rho = 0.439, READ) were all positively correlated with high *PRRX1* expression (Figure 7). Elevated *IL1RN* expression was significantly and positively correlated with neutrophils (Rho = 0.664, COAD; Rho = 0.534, READ) and myeloid dendritic cells (Rho = 0.514, COAD; Rho = 0.427, READ; Figure 7). All *P*-values were well below 0.001. These suggested the crucial role of *IL1RN* and *PRRX1* in the immune infiltration of CRC. Furthermore, analysis of *IL1RN* and *PRRX1* expression in CRC in relation to immunomodulators revealed that both are intimately involved in the regulation of immune regulatory processes and that *IL1RN* may be associated with immune escape. Expression of *IL1RN* was significantly associated with immunosuppressive agents, such as CD274 (Rho = 0.537, COAD), HAVCR2 (RHO = 0.515, COAD), PDCD1LG2 (RHO = 0.489, COAD), IL-10 (RHO = 0.489, READ), HAVCR2 (RHO = 0.508 READ), and PDCD1LG2 (RHO = 0.542, READ). *IL1RN* is also closely linked to immunostimulatory factors, as shown by CD86 (RHO = 0.53, COAD), TNFRSF9 (RHO = 0.495, COAD), CXCR4 (RHO = 0.487, COAD), CD86 (RHO = 0.502, READ), and CD80 (RHO = 0.512, READ; Figure 8). *PRRX1* showed stronger correlations, for example, with immunostimulatory factors, CD86 (RHO = 0.683, COAD; RHO = 0.674, READ), ENTPD1 (RHO = 0.661, COAD; RHO = 0.652, READ), TNFSF4 (RHO = 0.779, COAD; RHO = 0.782, READ), CXCR4 (RHO = 0.513, COAD), and TNFSF13B (RHO = 0.613, COAD; RHO = 0.625, READ) were significantly associated with *PRRX1* expression, as well as with immunosuppressive factors, TGFB1 (RHO = 0.573, COAD; RHO = 0.502, READ), KDR (RHO = 0.566, COAD), HAVCR2 (RHO = 0.685, COAD; RHO = 0.654, READ), TGFBR1 (RHO = 0.529, COAD; RHO = 0.520, READ), and PDCD1LG2 (RHO = 0.685, COAD; RHO = 0.646, READ) were similarly the same (Figure 9). All of the above results were statistically significant.

### 3.7. Result 7

**Figure 10:**
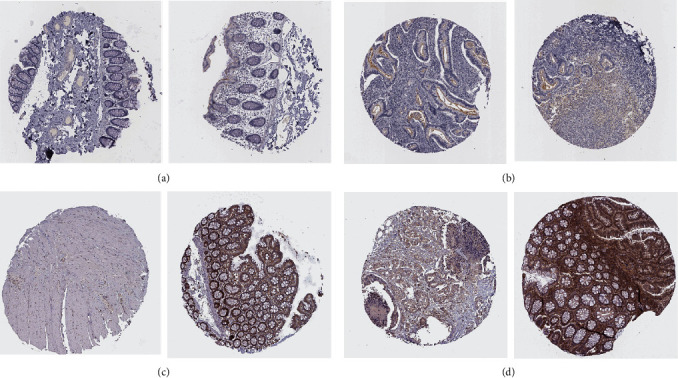
Immunohistochemical results of *IL1RN* and *PRRX1* in normal tissues and CRC tissues. (a) *IL1RN* in normal tissues. (b) *IL1RN* in CRC tissues. (c) *PRRX1* in normal tissues. (d) *PRRX1* in CRC tissues.

Elevated levels of *IL1RN* expression were correlated with antibody HPA001482, along with increased expression of *PRRX1* correlating with antibody HPA051084. Upon further analysis of the differences between *IL1RN* and *PRRX1* in normal colorectal tissues and CRC tissues, we found that *IL1RN* could not be detected in normal tissues, whereas in CRC tissues, *IL1RN* displayed weak staining (Figures 10(a) and 10(b)). *PRRX1* was also undetectable in normal colorectal tissues but presented high or medium staining in CRC tissues. However, the data in the HPA database failed to point out location of *PRRX1* concentration (Figures 10(c) and 10(d)).

## 4. Discussion

Mutations in multiple genes or somatic cells make a large part of causes that are associated with CRC heterogeneity. Prompt application of diagnostic markers for risk stratification and early detection would dramatically extend overall survival time [39]. There exists a phenomenon that contemporary diagnostic marker assays for CRC are undoubtedly shocking in number while disappointing in outcome [40]. Primarily, as the ideal screening or diagnostic biomarker is expected to be highly sensitive and specific, few similar markers fit this have been identified. Increasing attention is being paid to the role of immunotherapy on the curative side of CRC [41]. Meanwhile, studies noted that TILs were proved to be implicated in tumor immune responses, which might be a predictor of outcome in response to immunotherapy and prognosis [42, 43]. Identification of specific immune markers relevant to CRC and acquisition of new immunotherapeutic targets turns to be an imperative task.

Currently, by further analysis of 15 seed genes filtered out (IL1RN, GALNT12, ADH6, SCN7A, CXCL1, FGF18, SOX9, ACACB, PRRX1, MZB1, SLC22A3, CNNM4, LY6E, IFITM2, GDPD3), we discovered that IL1RN, ADH6, SCN7A, ACACB, MZB1, and GDPD3 were very limitedly studied in the context of CRC. Among them, *SCN7A*, *ACACB*, and *MZB1* showed statistically significant overall patient survival or disease-free survival. However, there exists an absence of literature related to these hub genes, and more studies on the mechanisms of CRC disease progression deserve to be given to these genes. Using GO and KEGG functional enrichment analyses, we found that hub genes are involved in tumor immunity in their functions, so we performed an immunobioinformatics database search of 15 key genes to identify potential immune infiltration marker roles of *IL1RN* and *PRRX1.*

*IL1RN* was the hub gene with the most edges found in this study, despite the fact that its overall survival or disease-free survival showed differences between the cancer-bearing and normal populations, but online databases suggested that the results were not statistically significant. Ma et al. had explored the ability of antagonizing IL-1 to inhibit CRC liver metastasis, but did not dive into the prospects of *IL1RN* in the context of CRC [44]. Wang et al. demonstrated that targeting the metabolism of amino acids like depriving methionine or targeting *IL1RN* might provide novel orientations in curing glioma [45]. *IL1RN* polymorphisms have similarly been proven to reduce the population risk of thyroid cancer risk [46]. Existing literature suggests that *IL1RN* is closely related to tumor immunity and tumor metabolism, of which further studies are needed to investigate its value in the prognosis of immune infiltration. According to our study, a strong correlation was shown between *IL1RN* and CD274, which is well-known as PD-L1 [47]. A brunch of evidences could confirm the role of PD1/PDL1, an essential component of immune checkpoints, in regulating TIL function [47]. CD274 participates widely in the resistance of various cancers to treatment, such as chemotherapy and targeted therapies as an important immunosuppressive factor [48]. Targeting immune checkpoint blockade of PD-1/PD-L1 is well established in diverse tumors, with targeted PD-L1 emerging as a routine treatment for common malignancies, including CRC [49]. The correlation between *IL1RN* and CD274 implies that *IL1RN* is likely to be involved in PD-L1 targeted therapy in the future. In addition, we noted *PRRX1* possessed the astonishing potential to be a prognostic biomarker correlated with immune infiltrates in CRC. Currently, a few publications have described the capacity of PRRX1 to induce the EMT process in CRC cells, which in turn facilitates distant CRC metastasis [50]. Moreover, studies of *PRRX1* in alternative tumor contexts also focus mostly on its induction of the EMT process in tumor cells, which leads to implications of proliferation, migration, and invasion of tumor cells [51, 52]. However, there exists little report on the potential of *PRRX1* to interact with tumor immune infiltration and thus affect patient prognosis. By compiling data from online database, we found a remarkable correlation between *PRRX1* and TILs. *PRRX1* showed significant correlations with both immunostimulators and immunoinhibitors. For instance, the correlation coefficient between *PRRX1* and CD86, a co-stimulatory molecule on antigen-presenting cells that had been demonstrated to act as a pivotal role in tumor immunity in pancreatic and bladder cancers, reached 0.683 [53, 54]. In addition, as the tumor pathology progressed, the expression of *PRRX1* in CRC tissues gradually increased. All these evidences pointed to the promising potential of *PRRX1* as a marker of tumor immune infiltration. Although current study initially reveals the utility of IL1RN and PRRX1 as markers of immune infiltration, what is lacking is that this conclusion relies only on data from online databases and lacks specific experiments to validate this unique potential.

Furthermore, although our study identified hub genes as affecting CRC and revealed promising potential for being biomarkers, subgroup information, such as tumor location as well as staging, was not precisely pinpointed. Along with the disclosure of increased sequencing data, precisely targeting biomarker function to location-specific, stage-specific features would be much more likely.

Similarly, whereas the aforementioned *SCN7A*, *ACACB*, and *MZB1* showed statistically significant prognostic results, further biological experiments are required to investigate their roles in the context of CRC.

## 5. Conclusion

In conclusion, 704 DEGs with specific expression in CRC were identified by bioinformatics means, basing on which 15 selected genes with enhanced differential properties were further identified, all of which may operate as diagnostic markers for CRC. Furthermore, 15 seed genes were further characterized to identify initially the potential of *IL1RN* and *PRRX1* as markers of tumor immune infiltration in CRC tissues.

## Figures and Tables

**Table 1:** Functional roles of 15 seed genes.

Gene symbol	Function
*IL1RN*	Regulating various immune as well as inflammatory reactions related to interleukin 1, specifically at the early stage of infectious or inflammatory conditions.
*GALNT12*	Facilitating the catalyzation of the transition of *N*- acetylgalactosamine from UDP-GalNAc to the surface acceptor at the first stage of *O*-linked protein glycosylation.
*ADH6*	Codifying for class V alcohol dehydrogenase belonging to the alcohol dehydrogenase family.
*SCN7A*	Encoding one of the many voltage-gated sodium channel proteins.
*CXCL1*	Acting as a chelating agent for neutrophils in the inflammatory process.
*FGF18*	Riching in functions regulating cell mitosis and maintaining cell survival activity, engaged in extensive biological processes including growth and invasion of diverse neoplasms.
*SOX9*	Intervening in the differentiation procedure of chondrocytes, and in concert with *SF1*, involved in the regulation of the transcribing of the *AMH* gene.
*ACACB*	Involving a pivotal step in the uptake and oxidation of fatty acids in mitochondria due to a mechanism involving inhibition of the ability of carnitine-palmitoyl-CoA transferase I to control the oxidation of fatty acids.
*PRRX1*	Enhancing the binding potential of serum response factors to induce cell growth and differentiation.
*MZB1*	Involved in positive regulation of cell population proliferation.
*SLC22A3*	Involved in the removal of numerous internal sources of small organic cations as well as various pharmaceutical substances or environmental toxins.
*LY6E*	Encoding a protein broadly engaged in regulating tumorigenesis and modulating immune function, which is located on the cell surface and supplies the anchor point for GPI.
*IFITM2*	The protein encoded by this gene restricts cellular entry by diverse viral pathogens.
*GDPD3*	Enabling phosphoric diester hydrolase activity. Involved in *N*-acylethanolamine metabolic process.
*CNNM4*	Encoding a protein that assists in the transport of metal ions.

## Data Availability

The data used to support and demonstrate the results of this investigation are available on the above-mentioned resources.

## References

[1] Sung H., Ferlay J., Siegel R. L. (2021). Global cancer statistics 2020: GLOBOCAN estimates of incidence and mortality worldwide for 36 cancers in 185 countries. *CA: A Cancer Journal for Clinicians*.

[2] Dekker E., Tanis P. J., Vleugels J. L. A., Kasi P. M., Wallace M. B. (2019). Colorectal cancer. *Lancet*.

[3] Alswealmeen W., Sadri L., Perrotti G. (2022). Colorectal cancer screening in the elderly: is age just a number?. *Clinical Colorectal Cancer*.

[4] Ladabaum U. (2022). Fulfilling the promise of colorectal cancer screening. *The Lancet Gastroenterology & Hepatology*.

[5] Grady W. M., Yu M., Markowitz S. D. (2021). Epigenetic alterations in the gastrointestinal tract: current and emerging use for biomarkers of cancer. *Gastroenterology*.

[6] Kanth P., Inadomi J. M. (2021). Screening and prevention of colorectal cancer. *BMJ*.

[7] Strickler J. H., Yoshino T., Graham R. P., Siena S., Bekaii-Saab T. (2022). Diagnosis and treatment of ERBB2-positive metastatic colorectal cancer: a review. *JAMA Oncology*.

[8] Wei F. Z., Mei S. W., Wang Z. J. (2020). Differential expression analysis revealing CLCA1 to be a prognostic and diagnostic biomarker for colorectal cancer. *Frontiers in Oncology*.

[9] Wang C., Fakih M. (2021). Targeting KRAS in colorectal cancer. *Current Oncology Reports*.

[10] Mulcahy M. F., Mahvash A., Pracht M. (2021). Radioembolization with chemotherapy for colorectal liver metastases: a randomized, open-label, international, multicenter, phase III trial. *Journal of Clinical Oncology*.

[11] Hu M., Wang Z., Wu Z. (2022). Circulating tumor cells in colorectal cancer in the era of precision medicine. *Journal of Molecular Medicine*.

[12] Taieb J., Kourie H. R., Emile J. F. (2018). Association of prognostic value of primary tumor location in stage III colon cancer with RAS and BRAF mutational status. *JAMA Oncology*.

[13] Duan L., Yang W., Wang X. (2019). Advances in prognostic markers for colorectal cancer. *Expert Review of Molecular Diagnostics*.

[14] Luo X. J., Zhao Q., Liu J. (2021). Novel genetic and epigenetic biomarkers of prognostic and predictive significance in stage II/III colorectal cancer. *Molecular Therapy*.

[15] Zaborowski A. M., Winter D. C., Lynch L. (2021). The therapeutic and prognostic implications of immunobiology in colorectal cancer: a review. *British Journal of Cancer*.

[16] Bai Z., Zhou Y., Ye Z., Xiong J., Lan H., Wang F. (2021). Tumor-infiltrating lymphocytes in colorectal cancer: the fundamental indication and application on immunotherapy. *Frontiers in Immunology*.

[17] Edgar R., Domrachev M., Lash A. E. (2002). Gene expression omnibus: NCBI gene expression and hybridization array data repository. *Nucleic Acids Research*.

[18] Lin G., He X., Ji H., Shi L., Davis R. W., Zhong S. (2006). Reproducibility probability score—incorporating measurement variability across laboratories for gene selection. *Nature Biotechnology*.

[19] Molnár B., Galamb O., Péterfia B. (2018). Gene promoter and exon DNA methylation changes in colon cancer development—mRNA expression and tumor mutation alterations. *BMC Cancer*.

[20] Galamb O., Spisák S., Sipos F. (2010). Reversal of gene expression changes in the colorectal normal-adenoma pathway by NS398 selective COX2 inhibitor. *British Journal of Cancer*.

[21] Meng J., Wei Y., Deng Q., Li L., Li X. (2022). Study on the expression of TOP2A in hepatocellular carcinoma and its relationship with patient prognosis. *Cancer Cell International*.

[22] Kestler H. A., Müller A., Gress T. M., Buchholz M. (2005). Generalized Venn diagrams: a new method of visualizing complex genetic set relations. *Bioinformatics*.

[23] Ashburner M., Ball C. A., Blake J. A. (2000). Gene ontology: tool for the unification of biology. The Gene Ontology Consortium. *Nature Genetics*.

[24] Kanehisa M., Goto S. (2000). KEGG: Kyoto Encyclopedia of Genes and Genomes. *Nucleic Acids Research*.

[25] Kanehisa M., Furumichi M., Tanabe M., Sato Y., Morishima K. (2017). KEGG: new perspectives on genomes, pathways, diseases and drugs. *Nucleic Acids Research*.

[26] Huang D. W., Sherman B. T., Tan Q. (2007). The DAVID Gene Functional Classification Tool: a novel biological module-centric algorithm to functionally analyze large gene lists. *Genome Biology*.

[27] Kanehisa M., Goto S., Kawashima S., Okuno Y., Hattori M. (2004). The KEGG resource for deciphering the genome. *Nucleic Acids Research*.

[28] Song W., Fu T. (2019). Circular RNA-associated competing endogenous RNA network and prognostic nomogram for patients with colorectal cancer. *Frontiers in Oncology*.

[29] Franceschini A., Szklarczyk D., Frankild S. (2013). STRING v9.1: protein-protein interaction networks, with increased coverage and integration. *Nucleic Acids Research*.

[30] Smoot M. E., Ono K., Ruscheinski J., Wang P. L., Ideker T. (2011). Cytoscape 2.8: new features for data integration and network visualization. *Bioinformatics*.

[31] Bandettini W. P., Kellman P., Mancini C. (2012). MultiContrast Delayed Enhancement (MCODE) improves detection of subendocardial myocardial infarction by late gadolinium enhancement cardiovascular magnetic resonance: a clinical validation study. *Journal of Cardiovascular Magnetic Resonance*.

[32] Maere S., Heymans K., Kuiper M. (2005). BiNGO: a Cytoscape plugin to assess overrepresentation of gene ontology categories in biological networks. *Bioinformatics*.

[33] Navarro Gonzalez J., Zweig A. S., Speir M. L. (2021). The UCSC genome browser database: 2021 update. *Nucleic Acids Research*.

[34] Ustjanzew A., Desuki A., Ritzel C. (2021). cbpManager: a web application to streamline the integration of clinical and genomic data in cBioPortal to support the molecular tumor board. *BMC Medical Informatics and Decision Making*.

[35] Chandrashekar D. S., Bashel B., Balasubramanya S. A. H. (2017). UALCAN: a portal for facilitating tumor subgroup gene expression and survival analyses. *Neoplasia*.

[36] Li T., Fu J., Zeng Z. (2020). TIMER2.0 for analysis of tumor-infiltrating immune cells. *Nucleic Acids Research*.

[37] Ru B., Wong C. N., Tong Y. (2019). TISIDB: an integrated repository portal for tumor-immune system interactions. *Bioinformatics*.

[38] Uhlén M., Fagerberg L., Hallström B. M. (2015). Proteomics. Tissue-based map of the human proteome. *Science*.

[39] Zygulska A. L., Pierzchalski P. (2022). Novel diagnostic biomarkers in colorectal cancer. *International Journal of Molecular Sciences*.

[40] Shaukat A., Levin T. R. (2022). Current and future colorectal cancer screening strategies. *Nature Reviews. Gastroenterology & Hepatology*.

[41] Fan A., Wang B., Wang X. (2021). Immunotherapy in colorectal cancer: current achievements and future perspective. *International Journal of Biological Sciences*.

[42] Zou Q., Wang X., Ren D. (2021). DNA methylation-based signature of CD8+ tumor-infiltrating lymphocytes enables evaluation of immune response and prognosis in colorectal cancer. *Journal for Immunotherapy of Cancer*.

[43] Turksma A. W., Coupé V. M., Shamier M. C. (2016). Extent and location of tumor-infiltrating lymphocytes in microsatellite-stable colon cancer predict outcome to adjuvant active specific immunotherapy. *Clinical Cancer Research*.

[44] Ma J., Liang W., Qiang Y. (2021). Interleukin-1 receptor antagonist inhibits matastatic potential by down-regulating CXCL12/CXCR4 signaling axis in colorectal cancer. *Cell Communication and Signaling: CCS*.

[45] Wang K., Liu H., Liu J. (2019). IL1RN mediates the suppressive effect of methionine deprivation on glioma proliferation. *Cancer Letters*.

[46] Li H., Wu Y., Zhao R. (2020). IL-1RN gene polymorphisms reduces thyroid cancer risk in Chinese Han population. *Molecular Carcinogenesis*.

[47] Fan Z., Wu C., Chen M. (2022). The generation of PD-L1 and PD-L2 in cancer cells: from nuclear chromatin reorganization to extracellular presentation. *Acta Pharmaceutica Sinica B*.

[48] Yuan Y., Adam A., Zhao C., Chen H. (2021). Recent advancements in the mechanisms underlying resistance to PD-1/PD-L1 blockade immunotherapy. *Cancers*.

[49] Hu X., Lin Z., Wang Z., Zhou Q. (2021). Emerging role of PD-L1 modification in cancer immunotherapy. *American Journal of Cancer Research*.

[50] Takahashi Y., Sawada G., Kurashige J. (2013). Paired related homoeobox 1, a new EMT inducer, is involved in metastasis and poor prognosis in colorectal cancer. *British Journal of Cancer*.

[51] Lv Z. D., Yang Z. C., Liu X. P. (2016). Silencing of Prrx1b suppresses cellular proliferation, migration, invasion and epithelial-mesenchymal transition in triple-negative breast cancer. *Journal of Cellular and Molecular Medicine*.

[52] Ma B., Ma J., Yang Y. (2020). Effects of miR-330-3p on invasion, migration and EMT of gastric cancer cells by targeting PRRX1-mediated Wnt/*β*-catenin signaling pathway. *Oncotargets and Therapy*.

[53] Xiang H., Zhao W., Sun Y. (2012). CD86 gene variants and susceptibility to pancreatic cancer. *Journal of Cancer Research and Clinical Oncology*.

[54] Tekguc M., Wing J. B., Osaki M., Long J., Sakaguchi S. (2021). Treg-expressed CTLA-4 depletes CD80/CD86 by trogocytosis, releasing free PD-L1 on antigen-presenting cells. *Proceedings of the National Academy of Sciences of the United States of America*.

